# A simple antegrade perfusion method for isolating viable single cardiomyocytes from neonatal to aged mice

**DOI:** 10.14814/phy2.13688

**Published:** 2018-04-26

**Authors:** Mariko Omatsu‐Kanbe, Kengo Yoshioka, Ryo Fukunaga, Hironori Sagawa, Hiroshi Matsuura

**Affiliations:** ^1^ Department of Physiology Shiga University of Medical Science Otsu Shiga Japan; ^2^ Department of Pediatrics Shiga University of Medical Science Otsu Shiga Japan

**Keywords:** Antegrade perfusion, isolation of cardiomyocytes, mouse heart

## Abstract

The aim of this study was to establish a simple and reproducible antegrade perfusion method for isolating single viable mouse heart cells and to determine the standard practical protocols that are appropriate for mice of various ages. Antegrade perfusion was performed by injecting perfusate from near the apex of the left ventricle of the excised heart, the aorta of which was clamped, using an infusion pump. This could thoroughly perfuse the myocardium through the coronary circulation. All procedures were carried out on a prewarmed heater mat under a microscope, which allows for the processes of injection and perfusion to be monitored. With appropriate adjustment of the size of the injection needle, the composition and amount of enzyme solution and the perfusion flow rate, this antegrade perfusion method could be applied to the hearts of neonatal to aged mice. We examined the morphological characteristics and electrophysiological properties of the isolated ventricular and atrial myocytes and found that these cells were mostly identical to those obtained with the traditional Langendorff‐based retrograde perfusion method. Interstitial nonmyocytes, such as cardiac progenitor cells, were also isolated simultaneously from the supernatant fraction of the centrifugation, similar to the retrograde perfusion method. The results suggest that single heart cells can be well isolated with high degree of quality by the present antegrade perfusion method, regardless of the age of the mouse.

## Introduction

The basic method of isolating single cells from dissected tissue is to mince the tissues into pieces and subsequently digest the extracellular matrix with enzymes (chunk method). In the heart, however, it is not easy to mince the robust myocardium, which includes large amounts of extracellular matrix components, such as collagen and elastin fibers. Furthermore, the fact that cardiomyocytes are highly sensitive to hypoxia, mechanical stress, low temperature and/or other changes in the microenvironment, makes it difficult to isolate viable cells with the chunk method. Thus, the retrograde perfusion of the coronary artery using the Langendorff‐based perfusion system (Langendorff 1898) has been utilized to digest the extracellular matrix and isolate viable cardiomyocytes from the mammalian heart (Berry et al. 1970; Powell et al. 1980). Further incubation of the isolated cells with high K^+^, Ca^2+^‐free medium at 4°C (Benndorf et al. 1985) or the addition of 10–20 mmol L^−1^ 2,3‐butanedione monoxime (BDM) to isolation solution (Zhou et al. 2000) has usually been conducted to reduce the ischemic damage to the cardiomyocytes during the isolation procedure.

In mouse models, Langendorff‐perfused isolated heart is also a reliable model for the analysis of the contractile functions and ischemia‐reperfusion responses (Reichelt et al. 2009). We have used a Langendorff‐based technique, using simple devices without the addition of BDM or nonphysiological chemicals to the solutions described by Shioya (2007), to isolate mouse ventricular myocytes (Omatsu‐Kanbe et al. 2010; Hoshino et al. 2012), atrial myocytes (Nakamura et al. 2010) and cardiac progenitor cells (atypically‐shaped cardiomyocytes, ACMs) (Omatsu‐Kanbe and Matsuura 2009). However, the cannulation of the mouse aorta and its subsequent mounting on the Langendorff‐apparatus to perform retrograde perfusion are delicate operations, requiring a high degree of skill. Thus, in addition to this retrograde perfusion method, it is desirable to develop more convenient techniques for obtaining single mouse heart cells. Recently, Ackers‐Johnson et al. (2016) reported a novel Langendorff‐free method for isolating cardiomyocytes from the adult mouse heart and analyzed the cellular function precisely. This new method is epoch‐making based on the antegrade perfusion of the coronary arteries.

In this study, we refined the reproducible antegrade perfusion method for isolating viable single heart cells. We also established the practical standard cell isolation protocols for the hearts of mice of all ages. The results show that the isolated heart cells are mostly comparable to those prepared by the Langendorff‐based retrograde perfusion method.

## Methods

### Ethical approval

All animal experiments conformed to the Guide for the Care and Use of Laboratory Animals published by the US National Institutes of Health (NIH Publication No. 85‐23, revised 1996) and were approved by the institutional Review Board of the Shiga University of Medical Science Animal Care and Use Committee (approved no. 2017‐5‐15). The methods were carried out in accordance with the approved guidelines.

### Animals

C57BL/6J mice were purchased from Charles River Japan. The animals were fed ad libitum in a 12:12 h light‐dark cycles, and sucking infants were housed with their mothers according to the Guidelines for the Husbandry and Management of Laboratory Animals of Research Center for Animal Life Science at Shiga University of Medical Science.

### Solutions

Cell isolation buffer (CIB) contained (in mmol L^−1^) 130 NaCl, 5.4 KCl, 0.5 MgCl_2_, 0.33 NaH_2_PO_4_, 22 glucose, 50 *μ*U mL^−1^ bovine insulin, and 25 Hepes (pH adjusted to 7.4 with NaOH) (Shioya 2007). CIB‐EGTA contained CIB supplemented with 0.4 mmol L^−1^ EGTA. CIB‐Ca^2+^‐BSA contained CIB supplemented with 0.2% bovine serum albumin (BSA) and 1.2 mmol L^−1^ CaCl_2_. Enzyme‐mix solution (Enzyme‐mix) contained CIB supplemented with 1 mg mL^−1^ collagenase (Class 2, Worthington Biochemical, USA), 0.06 mg mL^−1^ trypsin (T‐8003, Sigma‐Aldrich), 0.06 mg mL^−1^ protease (P‐5147, Sigma‐Aldrich), and 0.3 mmol L^−1^ CaCl_2_. Tyrode solution (Tyrode) contained (in mmol L^−1^) 140 NaCl, 5.4 KCl, 1.8 CaCl_2_, 0.5 MgCl_2_, 0.33 NaH_2_PO_4_, 5.5 glucose, and 5.0 Hepes (pH adjusted to 7.4 with NaOH). Tyrode‐BSA contained Tyrode supplemented with 0.2% BSA, 100 mg mL^−1^ penicillin and 0.1 mg mL^−1^ streptomycin. K^+^‐rich pipette solution contained (in mmol L^−1^) 70 potassium aspartate, 50 KCl, 10 KH_2_PO_4_, 1 MgSO_4_, 3 ATP (magnesium salt), 0.1 GTP (dilithium salt, Roche Diagnostics GmbH), 5 EGTA and 5 HEPES (pH adjusted to 7.2 with KOH).

### Isolation of single ventricular and atrial myocytes from mice of various ages by antegrade perfusion method

Mice were killed by an overdose of sodium pentobarbital (>300 mg kg^−1^, *i.p*. injection) with heparin (8000 U kg^−1^) and the thoracic cavity was opened to expose the heart. The adult heart was quickly sucked with a 3.5‐mL soft plastic transfer pipette (#86.11771, Sarstedt, Germany), the tip of which was cut to approximately the size of the heart, and then excised using curved scissors (Shioya 2007). In mice of ≤4 weeks of age, the heart was directly excised using curved scissors.

**Figure 1:**
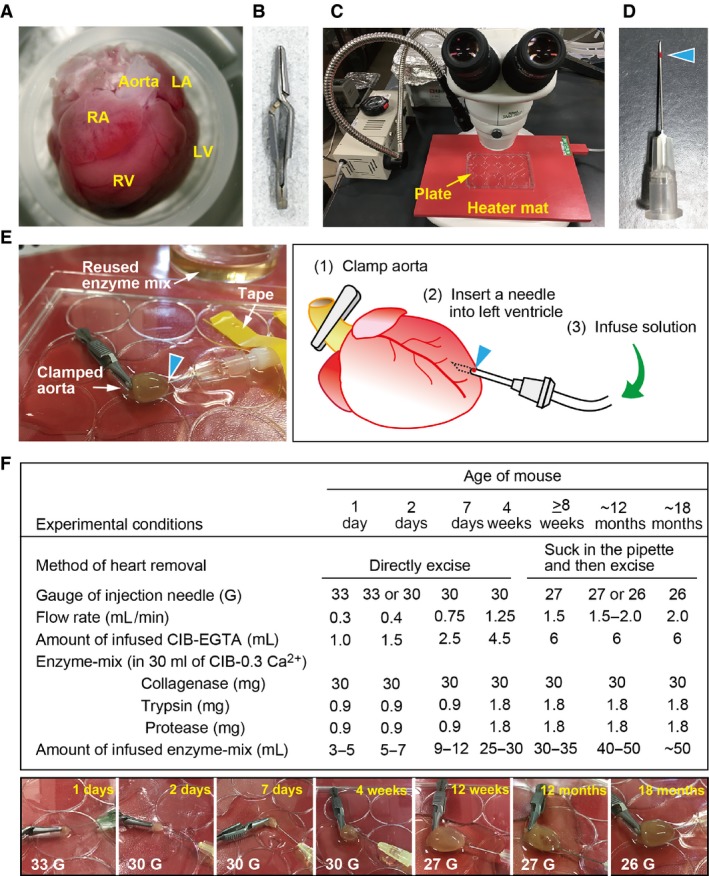
Protocols for the antegrade perfusion of the hearts of neonatal to aged mice. (A) The adult mouse heart placed in the heart‐stand (the lid of a 1.5‐mL sample tube) filled with CIB‐EGTA. LA, RA, LV, and RV indicate left and right atrium and ventricle, respectively. (B) A small straight vascular clamp (35 mm) used for clamping the aorta. (C) The instruments used for antegrade perfusion. Perfusion was performed on the lid of a plastic multi‐well culture plate placed on a prewarmed heater mat (red) under a stereoscopic microscope. The heater mat is for the heat retention of neonates, surgical operation, and postoperative warming of the mice constantly at 40°C. (D) An injection needle marked at 3 mm from the tip using red nail polish (arrowhead) to be a useful marker of the depth of the needle insertion. (E) Overview of the antegrade perfusion of the heart. The aorta was clamped and the needle (connected to the infusion tube) was inserted into near the apex of the left ventricle to a depth of within 3 mm using a red dot (arrowhead) as a depth marker. Thereafter, the needle was taped on the plate. Inject CIB‐EGTA to discharge blood and then enzyme‐mix successively using an infusion pump. The perfusion procedure was thus carried out on a prewarmed heater mat under a microscope. The cartoon shows the procedure of the antegrade perfusion of the excised heart. (F) Upper table shows the summary of the standard conditions for isolating heart cells from mice of various ages. Lower panels show that the hearts from neonatal to aged mice were antegradely perfused with enzyme‐mix on the prewarmed heater mat. The age of the mouse (yellow) and the gauge of the injection needle (white) are indicated in each panel. The same vascular clamp (B) is used for clamping the hearts of all ages.

The excised heart was immediately immersed in ice‐chilled CIB‐EGTA to stop it contracting, placed upright in a heart‐stand filled with CIB‐EGTA (Fig. 1A) and the tissues around the aorta were removed under a microscope. The heart‐stand consisted of the lid of a 1.5‐, 0.5‐ or 0.2‐mL sample tube (according to the heart size), attached to the ∅ 60 mm plastic culture dish. The aorta was then clamped with a small straight vascular clamp (Fig. 1B), and the clamped heart was transferred on a perfusion plate (we preferred to use the lid of a plastic multi‐well culture plate) placed on a prewarmed heater‐mat (Natsume Seisakusho, Japan) (Fig. 1C) and covered with dropped CIB‐EGTA to prevent the heart from drying. All subsequent procedures were performed under microscope. An injection needle connected to the infusion pump through the flexible tube was then carefully inserted near the apex of the left cardiac ventricle. A site 3 mm from the tip of the injection needle was marked with red nail polish (Fig. 1D), which was a useful marker of the depth of the needle insertion. Thereafter, the needle was taped on the plate, CIB‐EGTA was infused, using an infusion pump, to discharge the blood and prevent clotting; the enzyme‐mix was then infused to digest the tissues (Fig. 1E). The standard conditions for isolating heart cells from mice of different ages are listed in Figure 1F (upper table). The same small vascular clamp (Fig. 1B) was used for the mouse of every age (Fig. 1F, lower panels).

After perfusion with the enzyme‐mix, the clamp and the injection needle were removed, and then the atria were excised and immersed in the CIB‐Ca^2+^‐BSA at 37°C (kept on the heater mat). For isolation of the ventricular myocytes, the ventricles were cut out at the level of approximately 2/3 from the ventricular apex, not including the atrioventricular valves, and transferred in reused enzyme‐mix supplemented with 0.2% BSA placed on the heater mat (Fig. 1E). The tissue was gently made into small pieces with scissors and forceps, and the cells were dispersed with gentle pipetting. The cell mixture was then filtered through a cell strainer with a 150‐*μ*m mesh to remove undigested tissue debris. Thereafter, the procedure of the centrifugation and raising Ca^2+^ concentration with CIB‐Ca^2+^‐BSA was the same as those described for Langendorff‐based method (Shioya 2007). After the final centrifugation, the cardiomyocytes were resuspended in Tyrode‐BSA and kept at 37°C. In mice of <7 days of age, the cells were resuspended and stored in CIB‐Ca^2+^‐BSA at 37°C. In isolating the atrial myocytes, the epicardium of the excised atria was carefully torn and the cells were dispersed by the pipetting with a small pipette until obtaining the desired amount of the cells in CIB‐Ca^2+^‐BSA.

### Culturing of atypically‐shaped cardiomyocytes

The cardiac myocyte‐removed fraction was dispersed in semi‐solid culture medium and cultured in high‐quality plastic dishes (*μ*‐Dishes; ibidi GmbH, Germany) (Omatsu‐Kanbe and Matsuura 2009). Beating ACMs were identified in the culture after ~5 days.

### Whole‐cell patch‐clamp recordings

Membrane currents were recorded using the standard whole‐cell patch‐clamp techniques in voltage‐clamp mode (Hamill et al. 1981) with an EPC‐8 patch‐clamp amplifier (HEKA, Germany). The patch electrodes were fabricated from a glass capillaries (outside ∅ 1.5 mm, inside ∅ 0.9 mm, Narishige Scientific Instrument Lab., Japan) using a horizontal microelectrode puller (P‐97, Sutter Instrument Co., USA). The resistance of the electrode ranged from 2 to 4 MΩ when filled with a K^+^‐rich pipette solution. An aliquot of isolated ventricular myocytes was transferred to a recording chamber mounted on the stage of an inverted microscope superfused with Tyrode at a rate of 1 mL min^−1^ at 36–37°C. Membrane currents were activated by depolarizing voltage‐clamp steps applied from a holding potential of −40 mV to test potentials of −120 through +50 mV in 10‐mV steps. Action potentials were recorded using the perforated patch‐clamp method with K^+^‐rich pipette solution containing 30 mg mL^−1^ amphotericin B (Wako Pure Chemical Industries, Japan) by applying current pulses of 5–10 msec in duration at a rate of 1 Hz via the patch electrode. The action potential duration (APD) was measured at 20%, 50% and 90% repolarization levels (APD_20_, APD_50_, and APD_90_, respectively).

### Immunostaining

Isolated cardiomyocytes were immobilized with CELL‐TAK (Corning), fixed with 4% paraformaldehyde in PBS and permeabilized/blocked with 0.2% Triton X‐100 and 10% fetal bovine serum (FBS) in PBS. The cells were then labeled with the primary antibody and probed with an Alexa Fluor 488‐ or 568‐conjugated secondary antibody (Molecular Probes‐Thermo Fisher). The nuclei were stained with 1 *μ*g mL^−1^ 4′,6‐diamidino‐2‐phenylindole (DAPI). The fluorescent signals were analyzed using C1Si confocal laser scanning system on an Eclipse TE2000‐E inverted microscope (Nikon). The data reflect representative images obtained from three experiments.

### Antibodies

The primary antibodies were mouse monoclonal anti‐α‐actinin (anti‐ACTN, A7811, clone EA‐53, 1:800; Sigma‐Aldrich) and rabbit polyclonal anti‐connexin 43 (anti‐Cx43, #3512 1:200; Cell Signaling Technology) and anti‐atrial natriuretic peptide (anti‐ANP, AB5490, 1:200; Chemicon‐Millipore) antibodies.

### Statistical analysis

The data are presented as the mean ± SD. Student's *t*‐test was used for the analyses, and *P* < 0.05 were considered to indicate statistical significance.

## Results

### The ventricular and atrial myocytes isolated from mice of various ages

**Figure 2:**
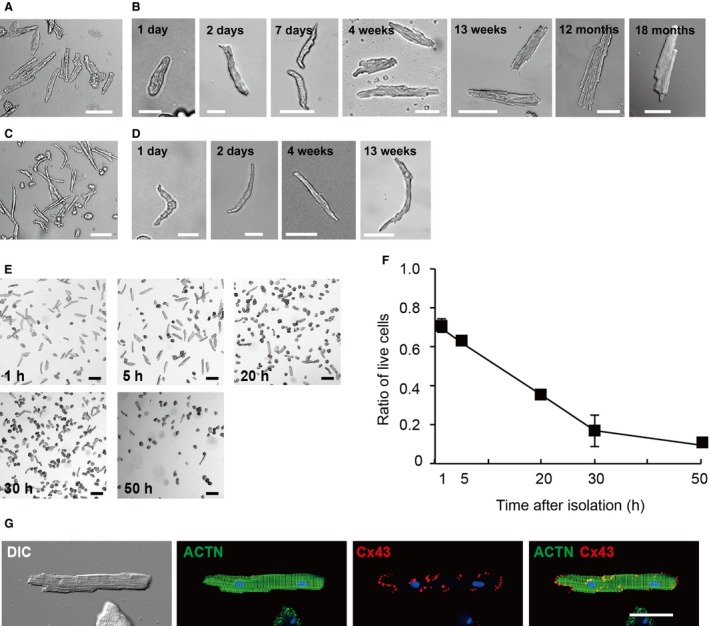
Morphology and storage life of the cardiac myocytes in Tyrode solution. (A, B) Representative images of ventricular myocytes from adult mouse (A), bar, 50 *μ*m. Ventricular myocytes from mice of 1, 2 and 7 days of age, 4 and 13 weeks of age, and 12 and 18 months of age (B). Bars, 1 and 2 days for 20 *μ*m; 7 days to 18 months for 50 *μ*m. (C, D) Representative images of atrial myocytes from adult mice (C), bar, 50 *μ*m. Atrial myocytes from mice of 1 and 2 days of age, and 4 and 13 weeks of age (D). Bars, 1 and 2 days for 20 *μ*m; 4 and 13 weeks for 50 *μ*m. (E) Ventricular myocytes stored in Tyrode‐BSA at 37°C for 1, 5, 20, 30, and 50 h after isolation. Bars, 150 *μ*m. (F) The time‐dependent changes in the viability of isolated ventricular myocytes during storage. Each value represents the mean±SD from three adult mice. (G) Confocal laser scanning microscopy of double‐immunostaining for ACTN (green) and Cx43 (red), DAPI staining for nuclei (blue) and DIC images of isolated adult ventricular myocytes. Bar, 50 *μ*m.

The overview and practical standard protocols of the antegrade perfusion for mouse of each age are demonstrated in Figure 1; the aorta of the excised heart was clamped, and then the perfusate was injected from near the apex of the left ventricle using an infusion pump. This procedure induced an enforcement of the flow in the coronary circulation, resulting in a thorough perfusion of the myocardium. The present antegrade perfusion method was found to be able to isolate heart cells from mice of all ages (from neonates to aged mice) by properly adjusting the size of injecting needle, the composition and amount of enzyme‐mix, and the flow rate of the injection pump. The time from sacrifice of the mouse until the final cell suspension was obtained was usually less than ~60 min for an adult heart and ~30 min for a neonatal heart, similar to the time required to perform the Langendorff‐based method. Using the appropriate protocols, we succeeded in isolating rod‐shaped ventricular (Fig. 2A, B) and spindle‐shaped atrial (Fig. 2C, D) myocytes from mice of various ages.

To examine the storage life of living adult ventricular myocytes, the isolated cells were suspended in Tyrode‐BSA and incubated for 1 to 50 h at 37°C. Cardiomyocytes suspended in Tyrode‐BSA were judged as living cells when the long axis of the cells was longer than twice as long as the shorter axis (Fig. 2E). Viability of the cardiomyocytes was defined as the percentage of the rod‐shaped cells in the cell count. The cell viabilities at 1, 5, 20, 30, and 50 h after isolation were 72.3 ± 2.4, 61.4 ± 1.8, 35.2 ± 1.3, 16.6 ± 7.9, and 9.5 ± 1.7%, respectively (Fig. 2F). The data were similar to those of adult mouse ventricular myocytes isolated by the Langendorff‐based retrograde perfusion method and stored in the same solution (Shioya 2007). Immunostaining analysis confirmed the expression of contractile protein *α*‐actinin (ACTN) and gap junction protein connexin 43 (Cx43) at the expected sites of ventricular myocyte (Fig. 2G).

### The electrophysiological properties of mouse ventricular and atrial myocytes

**Figure 3:**
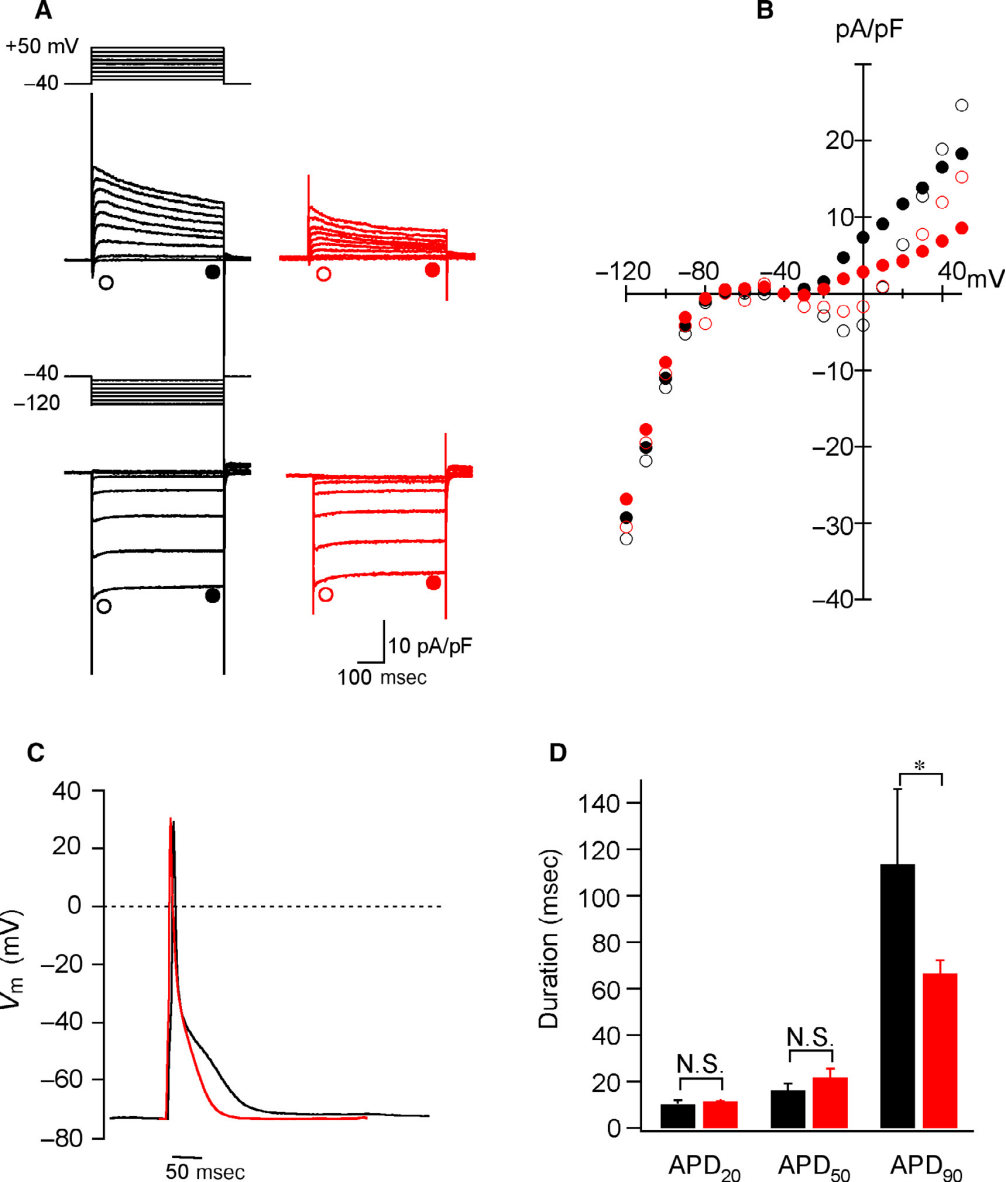
Electrophysiological properties of isolated mouse ventricular and atrial myocytes. (A) Representative superimposed current traces during 500‐ms voltage clamp steps applied from a holding potential of −40 mV to test potentials of −120 to +50 mV in 10‐mV steps recorded from isolated ventricular (left, black) and atrial (right, red) myocytes from adult mice. (B) Current‐voltage (I‐V) relationships for membrane currents measured at the initial (open circle) and end (closed circle) of 500‐msec voltage steps in ventricular (black) and atrial (red) myocytes shown in A. (C) Representative action potentials recorded from isolated ventricular (black) and atrial (red) myocytes of adult mice. (D) The action potential durations (APDs) recorded from ventricular (black column) and atrial (red column) myocytes. Each value represents the mean ± SD (*n* = 4). *P = 0.035.

The membrane currents of adult ventricular and atrial myocytes were recorded. Figure 3A represents the superimposed current traces during 500‐msec voltage clamp steps applied from a holding potential of −40 mV to test potentials of −120 to +50 mV in 10‐mV steps. The I‐V relationships of the membrane currents demonstrated that both the inward and outward currents of ventricular myocytes are larger than those in atrial myocytes (Fig. 3B). We then examined the action potentials of the cells under whole‐cell clamp condition (Fig. 3C). The APD20, APD50, and APD90 of ventricular myocytes were 10.3 ± 1.8, 16.2 ± 2.9, and 113.4 ± 32.6, while those of atrial myocytes were 11.4 ± 0.03, 21.8 ± 3.7, and 66.7 ± 5.5 msec, respectively (Fig. 3D).

### The isolation of beating atypically‐shaped cardiomyocytes (ACMs)

**Figure 4:**
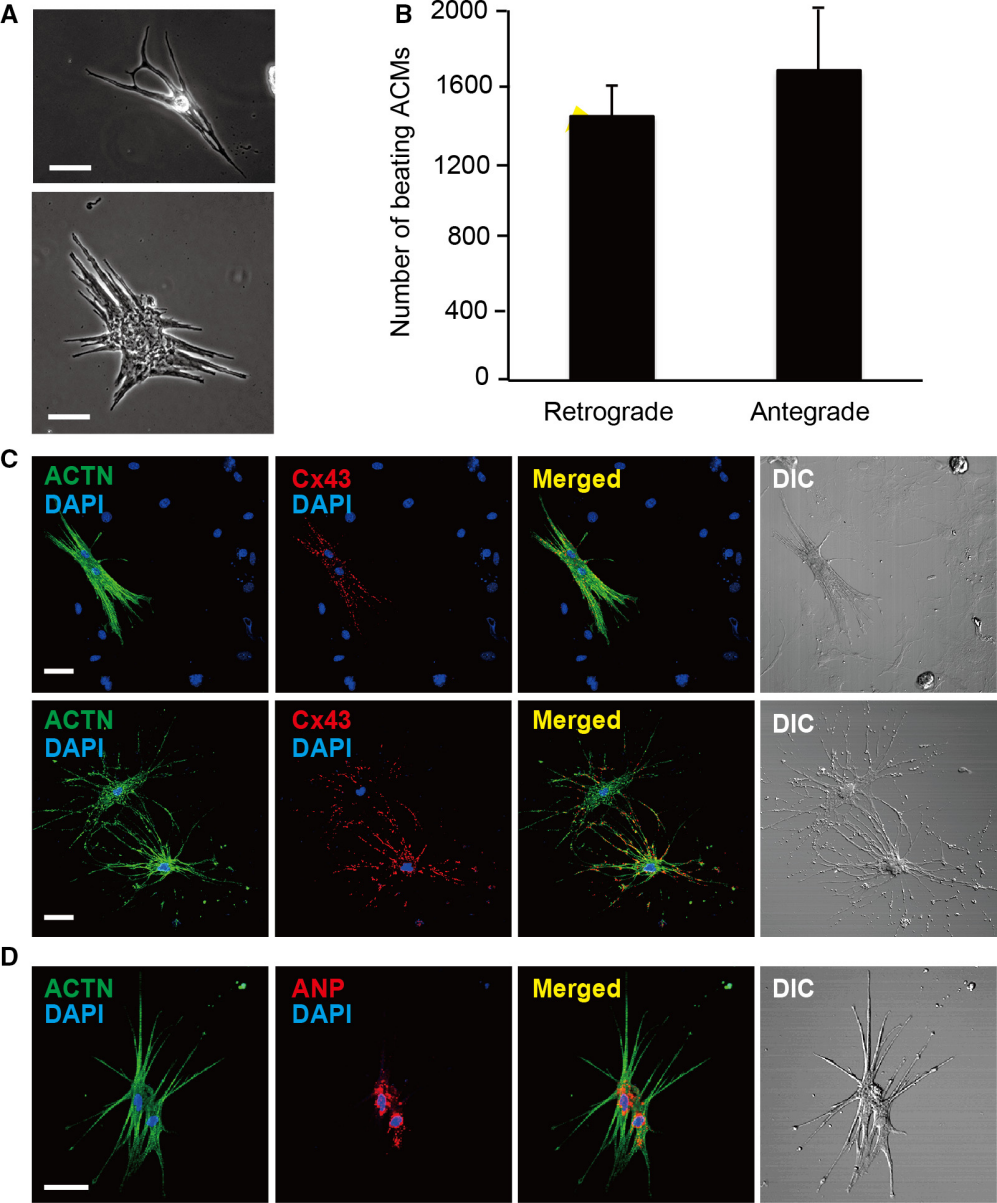
Preparation of atypically‐shaped cardiomyocytes (ACMs) from adult mice by the antegrade perfusion method. (A) Phase contrast images of beating ACMs cultured for 7–13 days. Bar, 50 *μ*m. (B) Cell numbers of beating ACMs cultured for 9 days obtained by the retrograde and the antegrade perfusion methods. Each value represents the mean±SD from three mice. The yields of cells obtained by the two methods did not differ to a statistically significant extent. *P* = 0.178. (C) Double‐immunostaining for ACTN (green) and Cx43 (red), DAPI‐staining (blue) and DIC images in solitary cell (upper) and two cells connected with the edges of the elongated plasma membrane (lower). Cultured for 7–13 days. Bar, 50 *μ*m. (D) Double‐immunostaining for ACTN (green) and ANP (red) in ACMs cultured for 13 days. Bar, 50 *μ*m. The cells were automatically beating before fixation.

ACMs are a type of cardiac progenitor cells identified in the cultures of cardiomyocyte‐removed fractions obtained from mouse cardiac ventricles that spontaneously develop into beating cells (Omatsu‐Kanbe and Matsuura 2009) with higher ischemic resistance in comparison to ventricular myocytes (Omatsu‐Kanbe and Matsuura 2013). Since native ACMs in the heart exist in the interstitial spaces among the ventricular myocytes (Omatsu‐Kanbe et al. 2014), the sufficient isolation of the cardiomyocytes is needed to obtain these cells. Beating ACMs were detected in the culture of cardiomyocytes‐removed fractions prepared with the antegrade perfusion method several days after plating (Fig. 4A). The numbers of beating cells obtained from the cardiac ventricles with the retrograde and antegrade perfusion methods (1428 ± 286 and 1673 ± 459, respectively) did not differ to a statistically significant extent (Fig. 4B). Immunostaining analysis confirmed the well‐organized sarcomeric structures detected by ACTN and the abundant expression of Cx43 at the plasma membrane in solitary (Fig. 4C, upper) and connected ACMs (Fig. 4C, lower) but not in the surrounding proliferating cells. The expression of atrial natriuretic peptide (ANP) was found to be at the perinuclear area in ACMs (Fig. 4D). These observations are identical to those in the cells obtained by the Langendorff‐based retrograde perfusion method (Omatsu‐Kanbe et al. 2010, 2017).

## Discussion

The present study demonstrates a simple and reproducible antegrade perfusion method for isolating single viable heart cells from neonatal to aged mice without the use of a Langendorff apparatus. Furthermore, in this method, the whole procedure can be observed under a microscope. We also establish the practical standard protocols of this method, which are appropriate for mouse of all ages, using isolation solutions without BDM, the same solutions as are used for Langendorff‐based method (Shioya 2007), to maintain the consistency of the previous data.

One of the biggest advantages of the antegrade perfusion method is that it reduces the technical obstacles associated with the Langendorff‐based retrograde perfusion method, and another is that it enables to thoroughly perfuse myocardium through the coronary circulation of the excised heart. To overcome the problems of the original antegrade perfusion method (Ackers‐Johnson et al. 2016), we improved each step of the procedure to raise the reproducibility of the manipulation of hearts from mice of all ages. The present antegrade perfusion procedure is carried out on the prewarmed heater mat under a microscope, which enables to see the needle insertion near the apex of the left ventricle and to keep the temperature of the heart during the perfusion. And whether the needle was inserted in the right place can be confirmed by the disappearance of the blood from the coronary arteries when the perfusion was started. In Langendorff‐based retrograde perfusion, the flow rate (under constant hydrostatic pressure) is accelerated during digestion and reaches more than twice the initial level, which indicates complete digestion. However, in antegrade perfusion, it is difficult to know when digestion is completed because the enzymes are constantly infused through the left ventricle by an injection pump. We therefore, established practical standard experimental conditions for neonatal to aged mice (Fig. 1). The total number of ventricular myocytes obtained per adult heart using this protocol was 2.3 × 10^6^±1.7 × 10^5^ cells (*n* = 3 mice), similar to the value previously reported, 2–3 × 10^6^ cells by Limana et al. (2002) and 3 × 10^6^ cells by Shioya (2007). This isolation procedure resulted in a high yield (~72%) of rod‐shaped quiescent ventricular myocytes from adult mice (Fig. 2F), similarly to that prepared by the traditional Langendorff‐based procedure (Shioya 2007). The present protocols used the isolation solution including trypsin, suitable for obtaining cells for the electrophysiological experiments, but may affect the cardiomyocyte survival in culture and storage life. It should be necessary to select the appropriate composition of the isolation solution depending on a purpose of the experiments.

The cell isolation method, chunk or perfusion method, affects some ionic currents in cardiomyocytes (Yue et al. 1996; Hoshino et al. 2012). Using Langendorff‐based retrograde perfusion method, the yield of rod‐shaped ventricular myocytes from neonatal mouse is low, but enough amount of rod‐shaped cardiomyocytes for applying patch clamp experiments (Hoshino et al. 2012). In the present antegrade perfusion method, the yields of rod‐shaped ventricular myocytes from mice of 7–14 days of age and 0–5 days of age were ~50% and ~20%, respectively (data not shown). The data were similar to those obtained by the Langendorff‐method using the same isolation solutions (Hoshino et al. 2012). Whether to choose “chunk or perfusion” or “retrograde or antegrade perfusion” method may depend on the purpose of the experiments.

The electrophysiological analyses demonstrated that the cardiomyocytes prepared by the present antegrade perfusion method can be used for whole‐cell patch clamp recording (Fig. 3). The data revealed that the amplitudes of the initial and end currents of 500‐ms voltage clamp steps in whole membrane currents were basically similar to those in cells obtained by the Langendorff‐based method (Nakamura et al. 2010; Hoshino et al. 2012). However, detailed experiments will be required to examine the functional similarity of these cells.

Cardiomyocytes are estimated to account for ~56% of the total cells in the mouse heart (Banerjee et al. 2007) and more recent studies suggest this number is ~30% (Pinto et al. 2016), indicating that nonmyocytes account for more than 40% of the heart cells. As demonstrated in the original report (Ackers‐Johnson et al. 2016), the antegrade perfusion method is suitable for the isolation of small cells, classified as nonmyocytes, which exist in the interstitial spaces among the cardiomyocytes. Since these cells are packed in small spaces or are tightly adhered to the basement membrane in the myocardium, the complete isolation of these cells is one of the barometers of the successful digestion of the extracellular matrix. ACMs are a type of cardiac progenitor cells in a diameter of 10 *μ*m when isolated, much smaller than cardiomyocytes, and develop into large beating cells (Fig. 4). The present antegrade perfusion method is thus useful for the isolation of various types of the cells from the mouse heart.

It is unlikely that this antegrade perfusion method can be applied in the isolation of cardiomyocytes from the hearts of rats or larger experimental animals. However, the present method may have the potential to become a new standard technique for isolating single heart cells in mice.

## Conflict of Interest

The authors declare no competing financial interests.
